# Loss of function of the carbon catabolite repressor CreA leads to low but inducer‐independent expression from the feruloyl esterase B promoter in *Aspergillus niger*

**DOI:** 10.1007/s10529-021-03104-2

**Published:** 2021-03-18

**Authors:** Jos Reijngoud, Mark Arentshorst, Claudine Ruijmbeek, Ian Reid, Ebru Demirci Alazi, Peter J. Punt, Adrian Tsang, Arthur F. J. Ram

**Affiliations:** 1 Molecular Microbiology and Biotechnology, Institute of Biology Leiden, Leiden University, Sylviusweg 72, 2333 BE Leiden, The Netherlands; 2 Centre for Structural and Functional Genomics, Concordia University, Montreal, Canada; 3 Dutch DNA Biotech, Hugo R Kruytgebouw 4-Noord, Padualaan 8, 3584 CH Utrecht, The Netherlands; 4 Present Address: Bioscienz, Goeseelsstraat 10, 4817 MV Breda, The Netherlands

**Keywords:** Aromatic compound, CreA, Ferulic acid, Hydroxycinnamic acid, Luciferase reporter, Plant cell wall

## Abstract

**Objective:**

With the aim to decipher the mechanisms involved in the transcriptional regulation of feruloyl esterase encoded by *faeB*, a genetic screen was performed to isolate *A. niger* mutants displaying inducer-independent expression from the *faeB* promoter.

**Result:**

*PfaeB-amdS* and *PfaeB-lux* dual reporter strains were constructed and used to isolate trans-acting mutants in which the expression of both reporters was increased, based on the ability to grow on acetamide plates and higher luciferase activity, respectively. The genetic screen on the non-inducing carbon source D-fructose yielded in total 111 trans-acting mutants. The genome of one of the mutants was sequenced and revealed several SNPs, including a point mutation in the *creA* gene encoding a transcription factor known to be involved in carbon catabolite repression. Subsequently, all mutants were analyzed for defects in carbon catabolite repression by determining sensitivity towards allyl alcohol. All except four of the 111 mutants were sensitive to allyl alcohol, indicating that the vast majority of the mutants are defective in carbon catabolite repression. The *creA* gene of 32 allyl alcohol sensitive mutants was sequenced and 27 of them indeed contained a mutation in the *creA* gene. Targeted deletion of *creA* in the reporter strain confirmed that the loss of CreA results in constitutive expression from the *faeB* promoter.

**Conclusion:**

Loss of function of CreA leads to low but inducer-independent expression from the *faeB* promoter in *A. niger.*

**Supplementary Information:**

The online version contains supplementary material available at 10.1007/s10529-021-03104-2.

## Introduction

As the most abundant organic carbon source available, plant biomass has a great potential to replace fossil fuels for renewable fuels and chemicals. Lignocellulosic biomass is considered to be the most favorable source for the production of second generation biofuels (Poovaiah et al. 2014). However, lignocellulosic biomass is highly recalcitrant to full and efficient enzymatic degradation by fungal enzyme cocktails. The recalcitrant nature of the lignocellulose is mainly derived from cell wall composition and architecture, and comprises different factors such as cellulose crystallinity, hemicellulose polymerization and substitution pattern, lignin content and composition, and the occlusion of the cell wall by lignin-hydroxycinnamate-hemicellulose cross-linking (McCann and Carpita 2015; Oliveira et al. 2015, 2019). In plant cell walls, ferulic acid (4-hydroxy-3-methoxycinnamic acid) plays a key role in inter- and intra-polymer cross-linking (Terrett and Dupree 2019).

Feruloyl esterases are a subclass of carboxylic esterases with the capacity to release ferulic acid and other hydroxycinnamic acids (such as *p-*coumaric acid) from plant cell wall. Because ferulic acid plays such a crucial role in crosslinking, feruloyl esterases play a key role to remove those cross-links and make the cellulose, hemicellulose and pectin accessible for hydrolysis by cellulases, hemicellulases and pectinases. Indeed, fungal feruloyl esterases have been shown to act synergistically with cellulases and xylanases to facilitate the degradation of complex plant cell wall biomass (Selig et al. 2008; Tabka et al. 2006; Wong et al. 2013).

*Aspergillus niger* is an important industrial producer of enzymes and an important model organism in relation to transcriptional regulation of plant cell wall degrading enzymes (de Vries et al. 2017; Pel et al. 2007). The genome of *A. niger* contains four genes encoding predicted feruloyl esterases (FaeA-FaeD) of which three (FaeA-FaeC) have been studied in detail (de Vries et al. 2002; Dilokpimol et al. 2017; Levasseur et al. 2004). Comparative analysis of their activities suggested that the different isoenzymes may target different substrates in a complementary manner (Dilokpimol et al. 2017). The expression of feruloyl esterases in *A. niger* is tightly regulated. Expression analysis of *faeA* revealed that the gene is specifically induced on xylose and arabinose (de Vries et al. 2002). Induction of *faeA* is dependent on the XlnR transcriptional regulator and co-expressed with xylanases and xylosidases (Gruben et al. 2017; van Peij et al. 1998). In addition, *faeA* was found to be induced on several aromatics including ferulic acid, vanillic acid, vanillyl alcohol, vanillin and veratric acid (de Vries et al. 2002). The *faeB* gene was not found to be induced by any carbon source tested, but was found to be strongly induced by aromatic compounds, such as ferulic acid, caffeic acid, and *p-*coumaric acid (de Vries et al. 2002; Dilokpimol et al. 2017). The expression of *faeC* was in general very low and induced by cinnamic acid (Dilokpimol et al. 2017). The induction pattern of *faeA, faeB* and *faeC* on various aromatic compounds suggests that their induction mechanism is mediated by different transcription factors and inducers.

Apart from being regulated via an induction mechanism, *faeB* was also shown to be sensitive for carbon catabolite repression in a CreA-dependent way (de Vries et al. 2002). CreA encodes a highly conserved C2H2 transcriptional repressor that represses the expression of enzymes allowing the preferential utilization of the energetically most favorable carbon source (Dowzer and Kelly 1991; Ries et al. 2016; Ruijter and Visser 1997).

We previously used forward genetic screens based on promoter-*amdS* reporter constructs, to isolate mutants showing constitutive expression of pectinase (Niu et al. 2017) and arabinase encoding genes (Reijngoud et al. 2019). To obtain insight into the transcriptional regulation of *faeB*, a similar strategy was used in which *PfaeB-amdS* and *PfaeB-lux* double reporter strains were constructed with the aim to identify mutants displaying constitutive, i.e. inducer-independent, expression from the *faeB* promoter. Using these reporter strains we show the specific induction of the *faeB* promoter by hydroxycinnamic acids. The mutants displaying inducer-independent expression from the *faeB* promoter were found to be defective in carbon catabolite repression.

## Materials and methods

### Strains, media and growth conditions

The *A. niger* strains used in this study are listed in Table 1. Strains were grown on liquid or solidified (containing 1.5 % (w/v) Scharlau agar) minimal medium (MM) or on complete medium (CM) as described (Arentshorst et al. 2012). *A. niger* transformants were isolated and purified as described (Arentshorst et al. 2012) using a final concentration of 100 µg/mL hygromycin or 20 µg/mL phleomycin. Mycelial growth assays were performed on acetamide plates, i.e. plates containing solidified MM with 10 mM acetamide as sole nitrogen source, 15 mM CsCl and 50 mM D-fructose, usually supplemented with 2.5 mM of the aromatic compound. Aromatic compounds were weighed, dissolved in sterile water and mixed with an equal volume of 2 × concentrated solidified MM. Radial growth of JR9.9 and JR9.19 was assayed by point inoculation of 5 µL filtered spore suspension (1 × 10^6^ spores/mL) in the center of an acetamide plate and incubation of the plates for 7 days at 30 °C. *Escherichia coli* DH5α was used for plasmid construction and cultured at 37 °C in lysogeny broth (LB) medium, with ampicillin (100 µg/mL).

**Table 1:** *A. niger* strains used in this study

Strain	Genotype	Reference
N402	*cspA*	ATCC® 64,974™ Bos et al. (1988)
AB4.1	*pyrG-* derivative of N402	van Hartingsveldt et al. (1987)
MA234.1	*ku70::PgpdA-amdS*	Alazi et al. (2016)
JR9.9	*PfaeB-amdS* in AB4.1	This study
JR9.19	*PfaeB-amdS* in AB4.1	This study
JR10.2	*PfaeB-lux in JR9.9*	This study
JR11.1	*PfaeB-lux in JR9.19*	This study
MA872.1	*ΔcreA::phleo in JR11.1*	This study

## Molecular techniques

PCR amplifications were performed using Phusion High-Fidelity DNA Polymerase (Thermo Scientific) according to the instructions provided. DNA fragments were purified using the GeneJET Gel Extraction Kit (Thermo Scientific) and ligations were carried out using the CloneJET PCR Cloning Kit and the Rapid DNA Ligation Kit (Thermo Scientific). DNA sequencing was performed by Macrogen.

## Plasmid construction

The primers used in this study are listed in Suppl. Table 1. Plasmid *PfaeB-amdS* was constructed as follows: the *A. niger faeB* promoter region (986 bp) was obtained by PCR using primers faeBP1f_NotI and faeABP2r and genomic DNA of N402 (Bos et al. 1988) as template. The 2.1 kb *A. nidulans amdS* gene was amplified using primers amdSP3f and amdSP4r_NotI and plasmid *PagsA-amdS-TamdS* (Damveld et al. 2008) as template. The overlap between faeABP2r and amdSP3f was used in a fusion PCR to obtain the 3.1 kb *PfaeB-amdS* fragment. The fragment was ligated into pJet1.2 (Thermo Scientific) and verified by sequencing to yield plasmid *PfaeB-amdS*.

Plasmid *PfaeB-lux*_*613*_ was constructed by PCR amplification of the *faeB* promoter using primers PfaeB_PmeI_P5f and PfaeB_PmeI_P6r using genomic DNA of *A. niger* strain N402 as a template, and subsequent cloning of the fragment in pJet2.1 to give pJet2.1_*PfaeB*. The 1 kb *faeB* promoter fragment from pJet2.1_*PfaeB* was obtained after *Pme*I digestion and cloned into *Pme*I digested pHT01. pHT01 contains a red firefly luciferase (λ_max_ = 613 nm) variant, codon-optimized for *A. niger*, containing a unique *Pme*I site to insert promoter fragments in front of the *lux*_*613*_ gene (Hein Trip, unpublished vector). After ligation, the *PfaeB-lux*_*613*_ plasmid was verified by sequencing.

The *creA* gene was deleted with the split marker method (Arentshorst et al. 2015), using phleomycin as selection marker. *creA* flanks were amplified using genomic DNA of *A. niger* strain N402 as template and primers creA_sm_P1f and creA_sm_P2r (*creA* 5’, 866 bp) or primers creA_sm_P3f and creA_sm_P4r (*creA* 3’, 778 bp). Phleomycin fragments were amplified using pAN8.1 (Punt and Van den Hondel 1992) as template and primers phleoP4f and phleoP8r (*phleo* 5’, 1143 bp) or primers phleoP6f and phleoP5r (*phleo* 3’, 1069 bp). *creA-phleo* 5’ split marker fragment (1991 bp) was obtained with fusion PCR, using fragments *creA* 5’ and *phleo* 5’ as template and creA_sm_P1f and phleoP8r as primers. *creA-phleo* 3’ split marker fragment (1826 bp) was obtained with fusion PCR, using fragments *creA* 3’ and *phleo* 3’ as template and phleoP6f and creA_sm_P4r as primers. Both split marker fragments were column-purified before transformation to *A. niger* strain JR11.1.

## Fungal transformations

Plasmid pJet1.2_*PfaeB-amdS* was introduced to *A. niger* strain AB4.1 by co-transformation with pAB4.1 (van Hartingsveldt et al. 1987). Transformants were purified on solidified MM without uridine and subsequently tested for growth on acetamide plates with or without 0.05 % (w/v) ferulic acid. Strain JR9.9 and JR9.19 were selected for their ability to grow on acetamide plates with ferulic acid, indicating that these transformants also had taken up the *PfaeB-amdS* plasmid. Strains JR9.9 and JR9.19 were subsequently transformed with *PfaeB-lux*_*613*_ by co-transformation with pAN7.1 (Punt et al. 1987). Purified transformants were analyzed for luciferase expression under non-inducing conditions (without ferulic acid or other aromatic compounds) or after addition of 0.05 % ferulic acid. JR10.2 (derived from JR9.9) and JR11.1 (derived from JR9.19) showed low luciferase activity under non-inducing conditions and induction of luciferase activity upon supplementation of the medium with ferulic acid (see also the Results section). The *creA* gene was deleted in JR11.1 using the split marker method with phleomycin as a selection marker (Punt and Van den Hondel 1992; Arentshorst et al. 2015) and verified by diagnostic PCR using primers listed in Supplementary Table 1.

## UV mutagenesis

UV mutagenesis experiments were performed as described previously (Damveld et al. 2008) using JR10.2 or JR11.1 as starting strains. After determining the killing rate of the UV treatment, spores with 60–70 % survival were plated out on acetamide plates without ferulic acid. On each plate about 5 × 10^5^ spores (50 µL of 1 × 10^7^ spores/mL suspension) were plated out. In total, around 10 plates for JR10.2 and 60 plates for JR11.1 were prepared and incubated for 7 days at 30 °C. Mutants growing and sporulating on the acetamide plates were purified twice on acetamide plates. Mutants with improved growth on acetamide plates were tested in the luciferase assay to identify trans-acting mutants.

## Luciferase assays

Luciferase (lux) assays were performed as follows: 176 µL of MM (containing 1 % (w/v) D-fructose and 0.003 % (w/v) yeast extract), 4 µL 25 mM luciferin (Promega, E1605) and 20 µL spore suspension (1*10^6^ spores/mL) were pipetted together (in triplicate) into a well of a white, clear bottom, 96 wells plate (Greiner Bio-One, ref. 655,095) and incubated for 24 h at 30 °C in the Spark 10 M Multimode Microplate reader (Tecan) while measuring luciferase activity and OD_600_ every 15 min. MM containing 0.1 % (w/v) ferulic acid was prepared by dissolving 0.25 g ferulic acid in 250 mL MM, followed by filter sterilization. MM with lower concentrations of ferulic acid was prepared by diluting MM containing 0.1 % ferulic acid with appropriate volumes of MM.

### Allyl alcohol sensitivity assay

Sensitivity towards allyl alcohol was determined by inoculating spores (5 µL of 1*10^6^ spores/mL) on plates containing MM with D-fructose and increasing concentrations of allyl alcohol (20 mM, 40 mM and 60 mM). Plates were incubated for 7 days at 30 °C before scoring the ability to grow on plates containing allyl alcohol.

### Genome sequencing and ***creA*** sequencing

Genomic DNA of *A. niger* strains was isolated as described (Arentshorst et al. 2012). The genomic DNA of strains JR10.2 and UV mutant JR10.2U#1 was further column-purified (NucleoSpin Plant II, Macherey-Nagel) for whole genome sequencing. The genomes of JR10.2 and JR10.2U#1 were sequenced at the McGill University Quebec Innovation Centre (QC, Canada) using the Illumina HiSeqX platform to about 50-fold coverage. Mapping of DNA reads to the NRRL3 genome was done using Bowtie2 (Langmead and Salzberg 2012). Sequence differences were detected with Freebayes (Garrison and Marth 2012). The DNA reads described in this study are deposited in the Sequence Read Archive under accession number PRJNA623364.

Sequencing of the *creA* gene of 32 mutants was performed by PCR amplification of the *creA* gene including 110 bp promoter and 128 bp terminator regions using the genomic DNA of the mutant as template DNA and primers p2f_creA_dPCR and p3r_creA_dPCR (Suppl. Table 1). Both primers were also used for subsequent sequencing, together with primer creA_rev.

## Results

### Construction and analysis of promoter reporter strains to assess *faeB* promoter activity in vivo

With the aim to isolate mutants showing constitutive expression from the *faeB* promoter, reporter strains were constructed containing the *PfaeB*-*amdS* and P*faeB-lux* reporters (Table 1). Since both reporters were introduced by co-transformation in which the site of integration or the copy number of the constructs is not known, two independent transformants (JR10.2, derived from JR9.9 and JR11.1, derived from JR9.19) were analyzed to make sure the growth phenotype or reporter activity was not influenced by these factors. In all experiments described below, both reporter strains responded similarly. The specificity of activation or repression of the *faeB* promoter by various aromatic compounds or carbon sources was tested in reporter strains JR9.9 and JR9.19 (both containing only the *PfaeB*-*amdS* reporter construct). Strains N402 and MA234.1 were included as negative and positive control, respectively. N402 does not contain the *A. nidulans amdS* gene and therefore cannot grow well on acetamide plates, i.e. solidified MM with acetamide as the sole nitrogen source, while MA234.1 contains the *amdS* gene expressed from the constitutive *gpdA* promoter and can therefore grow on acetamide plates. Strain MA234.1 was included also to assess possible toxic effects of the aromatic compounds which could interfere with the induction.

**Fig. 1:**
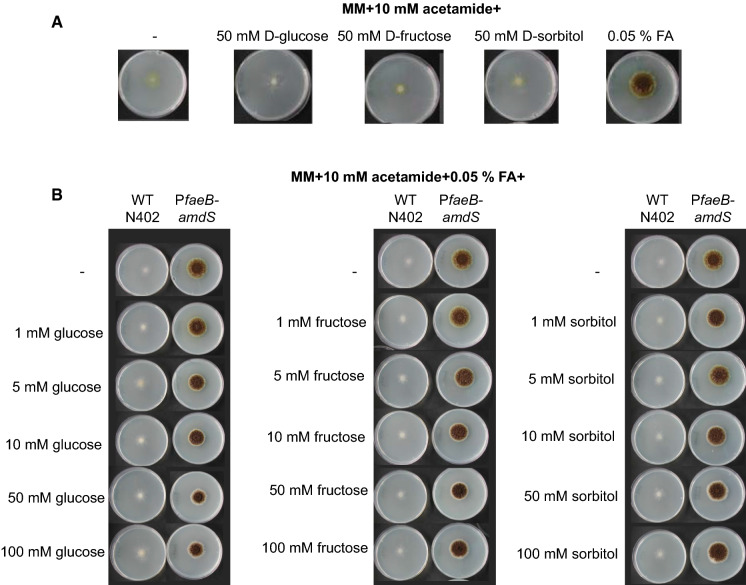
Growth analysis of *A. niger PfaeB-amdS* reporter strain (JR9.9). Expression from the *faeB* promoter is monitored in the *PfaeB-amdS* reporter strain by analyzing growth on MM with 10 mM acetamide and **a** 50 mM of different carbon sources or 0.05 % ferulic acid (FA); **b** 0.05 % FA and increasing concentrations of D-glucose, D-fructose, and D-sorbitol. Strains were grown for 7 days at 30 °C

The reporter strains JR9.9 and JR9.19 were not able to grow on acetamide plates without any carbon source or with D-fructose, D-glucose or D-sorbitol as the carbon source. Addition of 0.05 % ferulic acid resulted in growth of the reporter strains, showing the requirement for the presence of a specific inducer for the expression of *amdS* from the *faeB* promoter (Fig. 1a). Carbon sources, such as D-glucose, D-fructose and D-sorbitol, have been previously shown to impose CreA dependent repression on the promoters of genes encoding plant cell wall degrading enzymes including *faeB* (de Vries et al. 2002). The level of carbon catabolite repression for other promoters of pectinolytic genes such as *pgaX* and *pgxB* has also been shown to be dependent on the identity of the carbon source and the promoter (Alazi et al. 2018; Niu et al. 2015). Carbon catabolite repression on *faeB* promoter imposed by different carbon sources was assessed by adding varying concentrations of D-glucose, D-fructose or D-sorbitol (1 mM-100 mM) in acetamide plates containing 0.05 % ferulic acid. Presence of these carbon sources did not affect the growth of the reporter strains JR9.9 and JR9.19, indicating that the expression of *amdS* is at sufficiently high levels to enable growth since the *faeB* promoter is highly active in the presence of the inducer (ferulic acid) even when possibly repressing carbon sources are present (Fig. 1b).

**Fig. 2:**
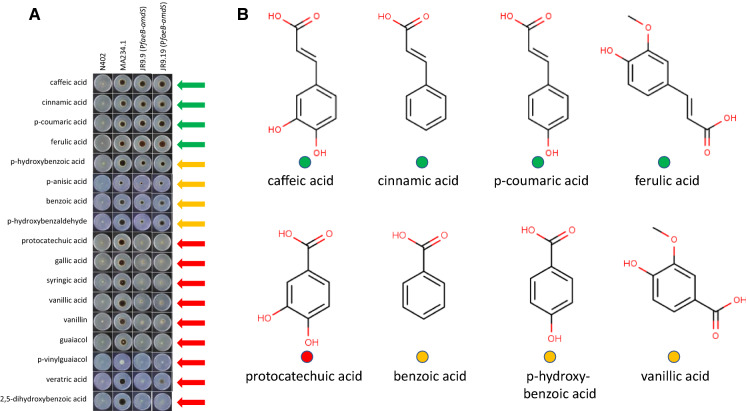
Expression from the *faeB* promoter is induced by various aromatic compounds. **a** Spores of reporter strains JR9.9 and JR9.19 and of control strains N402 and MA234.1 were inoculated on MM containing 1 % D-fructose, 10 mM acetamide and 2.5 mM of one of the different aromatic compounds. Pictures were taken after incubation for 7 days at 30 °C. The green, orange or red arrows next to the plates indicate high, moderate or low expression from the *faeB* promoter, respectively. **b** Chemical structures of nine of the aromatic compounds tested. The green, yellow or red dots, represent relative high, moderate or low induction of expression from the *faeB* promoter

Various aromatic compounds were added (final concentration 2.5 mM) to acetamide plates containing D-fructose and the ability of the reporter strains JR9.9 and JR9.19 to grow was analyzed (Fig. 2a). Fructose was used because it is considered as a non-inducing, non-repressing carbon source. As shown in Fig. 2a, four of the 17 aromatic compounds induced expression from the *faeB* promoter which allowed the reporter strain to grow on acetamide plates. These four compounds are ferulic acid, caffeic acid, cinnamic acid and *p-*coumaric acid. Four other compounds (*p-*hydroxybenzoic acid, *p-*anisic acid, benzoic acid and *p-*hydroxybenzaldehyde) moderately induced the expression of *amdS* via the *faeB* promoter. The addition of 2.5 mM *p-*anisic acid or *p-*vinylguaiacol reduced the growth of MA234.1, indicating that these compounds are toxic at this concentration. These results are largely comparable to the results described by de Vries et al. in which the expression of *faeB* was examined by Northern blot analysis after growing cells for two hours after transfer into medium containing various aromatic compounds (de Vries et al. 2002). Similar to our results, caffeic acid, *p-*coumaric acid and ferulic acid gave the strongest induction. In the de Vries study, weak induction of *faeB* expression by cinnamic acid was observed. In the present study, the robust growth of the reporter strain on acetamide suggests strong induction of *faeB* expression by cinnamic acid. *p-*Hydroxybenzoic acid was moderately inducing in both studies, while protocatechuic acid was inducing in the de Vries study, but not in ours. The differences might be explained by the different methods of measuring induction (Northern blot vs. growth readout via pFaeB-controlled AmdS reporter gene expression), the time of exposure to the compounds (induction after two hours vs. induction over days), or with the purity of the compounds. Comparison of the structures of the 17 different compounds (Fig. 2b and Supplementary Fig. 1) shows that the strongly inducing compounds all have a C3-moiety (prop-2-enoic acid (-C=C-COOH)) attached to the aromatic ring structure, suggesting that this C3-moiety is essential for activation of the *faeB* promoter. This specificity is also apparent from the observation that vanillic acid, *p-*hydroxy-benzoic acid, benzoic acid and protocatechuic acid, which have an identical aromatic ring structure as ferulic acid, *p-*coumaric acid, cinnamic acid, and caffeic acid respectively; but a C1 moiety (COOH) attached to the aromatic ring (Fig. 2b) do not induce high expression. The four moderately inducing compounds (*p-*hydroxybenzoic acid, *p-*anisic acid, benzoic acid and *p-*hydroxybenzaldehyde) lack the C3-moiety but share with the strongly inducing compounds the property that allylic side chains are absent (benzoic acid) or only found in the R4/para position on the aromatic ring. Additional allylic chains e.g. at the R3 position in protocatechuic acid decrease induction (Fig. 2b and Supplementary Fig. 1).

**Fig. 3:**
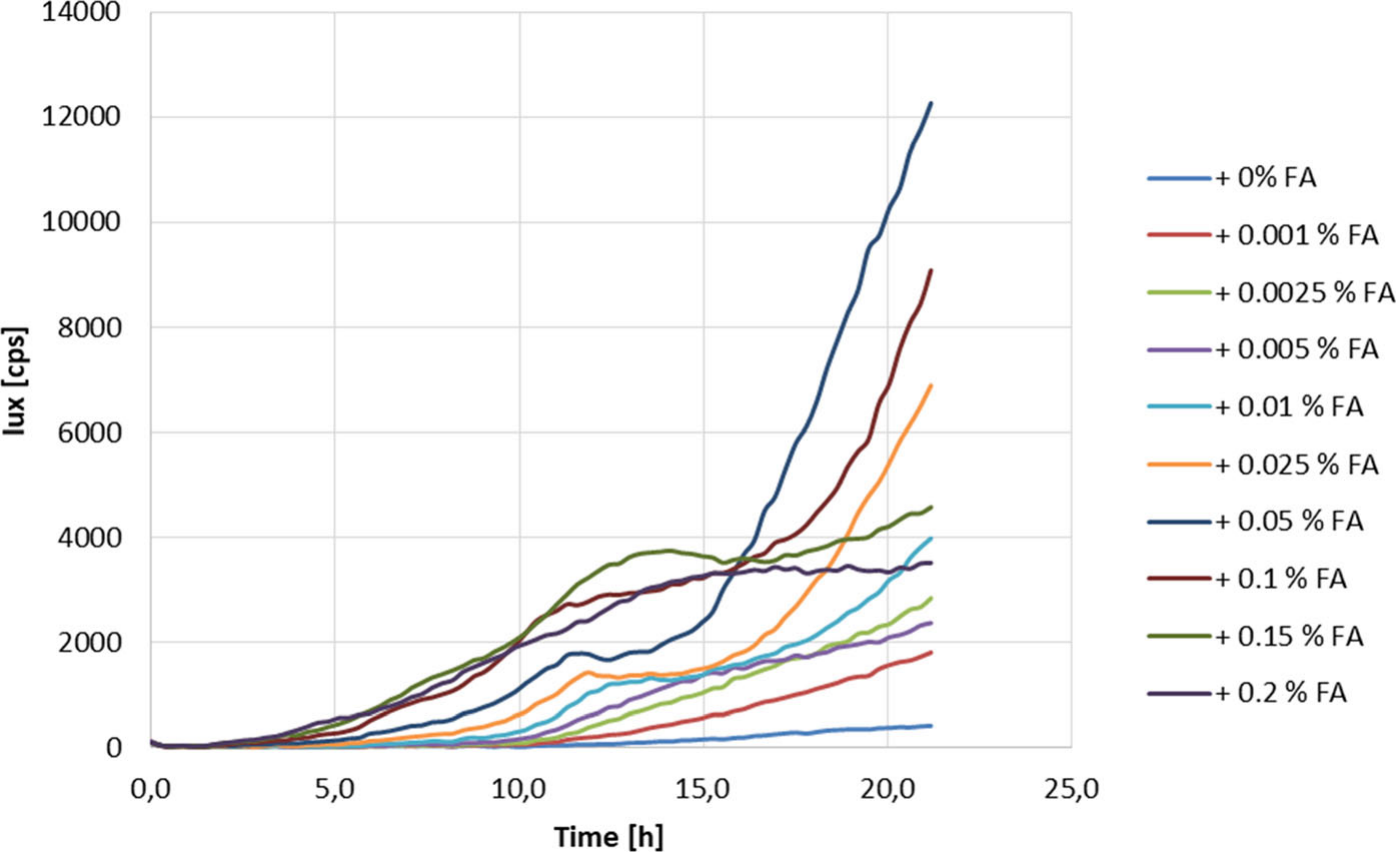
Expression of P*faeB-lux* is induced by ferulic acid in a concentration dependent manner. Expression of the *lux* reporter gene in strain JR10.2 was measured in a lux assay, using MM with D-fructose as carbon source with the addition of different concentrations (0 %-0.2 %) of ferulic acid (FA). Growth and luciferase activity were measured every 15 min for 24 h at 30 °C

The ability of ferulic acid to induce gene expression from the *faeB* promoter was also analyzed in *PfaeB-lux* reporter strains JR10.2 and JR11.1 by measuring luciferase activity during the first 24 h of growth. For this analysis, spores were inoculated in MM containing D-fructose with or without ferulic acid as inducing compound. As shown in Fig. 3 and Supplementary Fig. 2, the addition of ferulic acid induces luciferase activity in both reporter strains (JR10.2 and JR11.1) in a concentration-dependent way. The highest induction after 24 h was obtained when spores were germinated in the presence of 0.05 % (2.6 mM) ferulic acid (approximately 12,000 cps). Higher concentrations resulted in higher lux levels but had an inhibitory effect on the growth, resulting in lower lux levels after 24 h. Under non-inducing conditions, an approximately 20 times lower luminescence signal (approximately 500 cps) was detected after 24 h. This reflects the basal expression of luciferase from the *faeB* promoter under non-inducing conditions.

### lsolation of trans-acting mutants displaying inducer independent expression from the *faeB* promoter

**Fig. 4:**
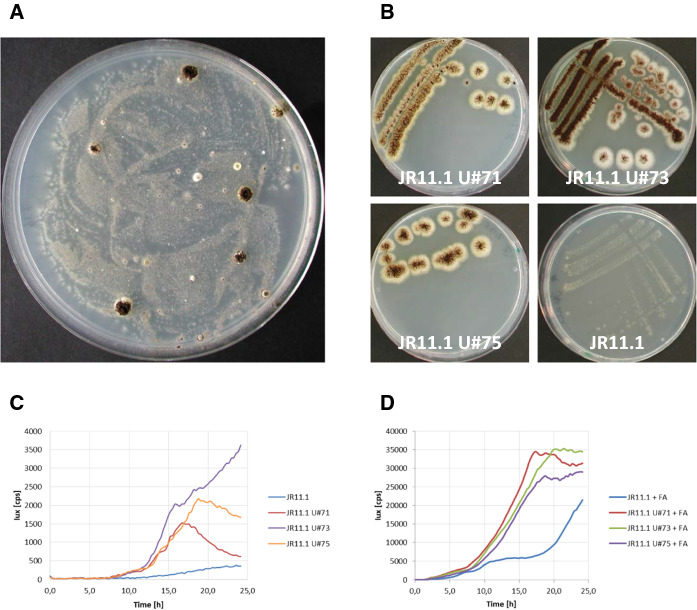
UV mutagenesis of JR11.1 results in mutants that show ferulic acid independent expression of *amdS* and *lux*. Spores of reporter strain JR11.1 were exposed to a series of UV light doses, and the survival rate after each dose was determined. **a** Spores with a survival rate of ± 60 % were plated out on selective medium (1 % D-fructose + 10 mM acetamide). **b** Mutants that were able to grow were purified on selective medium. **c **and **d** Expression of the second reporter gene *lux* was measured in a lux assay, using D-fructose as carbon-source without (**c**) or with the addition of ferulic acid (FA) (D). Growth and luciferase activity were measured every 15 min for 24 h at 30 °C

To obtain mutants with constitutive expression from the *faeB* promoter, spores of reporter strains JR10.2 and JR11.1 were UV-mutagenized (60–70 % survival rate) and plated out on acetamide plates containing D-fructose. Mutants that were able to grow and sporulate well on these plates were purified twice on acetamide plates containing D-fructose (Fig. 4a and b). After the plate screening, 13 strains in the JR10.2 background and 114 strains in the JR11.1 background were tested for luciferase activity to determine whether the mutations had a positive effect on both reporters expressed from the *faeB* promoter and are therefore trans-acting. All 13 mutants in the JR10.2 background and 98 mutants in the JR11.1 background showed > 2.5-fold higher luciferase activity compared to their corresponding parental strains under non-inducing conditions. The 2.5-fold was set as an arbitrary threshold level to consider these mutants as trans-acting mutants (Fig. 4c and Supplementary Table 2). ln the absence of ferulic acid as inducer, a low luminescence signal (approximately 400 cps) was detected in reporter strains JR10.2 and JR11.1 after 20 h of growth (Fig. 4c and Supplementary Fig. 2). ln the trans-acting mutants, under non-inducing conditions, the luminescence values varied among the transformants but were in general between four and 10 times higher than their corresponding parental strains (approximately 2500 cps in JR11.1 U#73) (Fig. 4c and Supplementary Table 2). When the inducer was present, a luminescence value of approximately 10,000 cps was reached in reporter strains JR10.2 and JR11.1 after 20 h, showing a clear induction of about 20-fold compared to the luminescence under non-inducing conditions (Fig. 3 and Supplementary Fig. 2). ln the trans-acting mutants, the luminescence signal was three to five times higher in the presence of inducer (approximately 35,000 cps in JR11.1 U#73) compared to their corresponding parental strains (Fig. 4d and Supplementary Table 2). The results show that the expression from the *faeB* promoter was three to 10 times higher in the trans-acting mutants in the presence of D-fructose regardless of the presence of the inducer.

## Genome sequencing of JR10.2U#1

To identify the mutation(s) responsible for the higher expression of the reporter genes via the *faeB* promoter in JR10.2U#1, the genomes of this mutant and its parental strain JR10.2 were sequenced and compared to each other. We identified in total 66 SNPs (Suppl. Table 3) of which 14 were located in coding regions of annotated genes (Table 2). Of those 14 SNPs, the mutation in the gene encoding the carbon catabolite repressor protein CreA was considered most relevant to explore further. The G to A point mutation results in R (arginine) to C (cysteine) amino acid change at position 117 of the CreA protein. The R117 is located in the second C2H2 motif in the CreA protein and is therefore likely to affect the DNA binding of CreA, possibly leading to an inactive CreA protein.

**Table 2:** Single nucleotide polymorphisms identified in ORFs in JR10.2 U#1

Gene	Chromosome	Position	Description	JR10.2	JR10.2U#1	JR10.2	JR10.2U#1
				Change at DNA-level		Change at Protein level	
NRRL3_01879	chr_2_2	829,765	Hypothetical protein	A→	G	R→	G
NRRL3_02036	chr_2_2	1,345,440	Hypothetical protein	G→	T	G→	W
NRRL3_02212	chr_2_2	1,846,987	Glycerol-3-phosphate dehydrogenase [NAD(+)]	G→	T	F→	L
NRRL3_03761	chr_3_2	660,719	MFS-type transporter	A→	G	I→	V
NRRL3_03766	chr_3_2	674,141	Short-chain dehydrogenase/reductase	A→	G	L→	P
NRRL3_04117	chr_3_2	1,678,195	Ankyrin repeat domain-containing protein	T→	A	Y→	*STOP
NRRL3_05946	chr_4_2	2,766,869	Carbon catabolite repressor CreA	G→	A	R→	C
NRRL3_06062	chr_4_2	3,130,181	K Homology domain-containing protein	G→	A	T→	I
NRRL3_06809	chr_5_2	724,637	Alcohol dehydrogenase domain-containing protein	T→	C	F→	L
NRRL3_07149	chr_5_2	1,707,985	Hypothetical protein	A→	G	R→	G
NRRL3_08507	chr_6_1	3,452,789	Uncharacterized protein	C→	A	K→	N
NRRL3_10110	chr_7_2	2,632,138	Carboxylesterase family protein	CT →	C	AF →	Frame shift
NRRL3_11650	chr_8_2	2,869,882	Sas10/Utp3/C1D family protein	C→	T	E→	K
NRRL3_11661	chr_8_2	2,900,726	Cation efflux transporter family protein	GT →	AA	KF→	KI

**Fig. 5:**
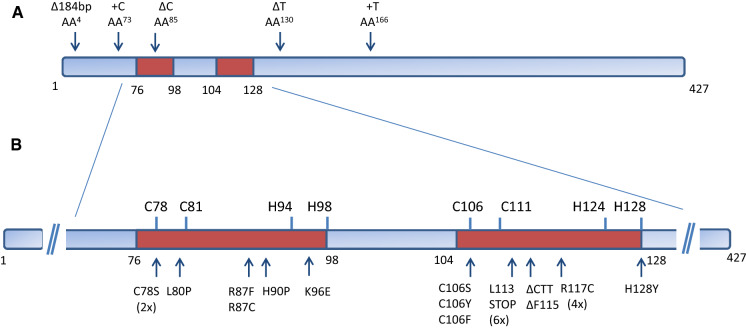
Mutations in CreA mainly occur in the C2H2 Zinc Finger DNA binding domains shown in brownish red. **a** Position in five mutants in which a deletions or insertion occurred. **b** Position and amino acid change in 22 mutants

To determine whether JR10.2U#1 and the other isolated mutants were mutated in the *creA* gene, first all mutants were tested for their sensitivity towards allyl alcohol. CreA represses alcohol dehydrogenase which converts allyl alcohol into acrolein, a toxic compound, and therefore *creA* loss-of-function mutants are more sensitive to allyl alcohol (Bailey and Arst 1975). All 13 mutants in the JR10.2 background and all 98 mutants in the JR11.1 background were more sensitive to allyl alcohol. From these results we concluded that our screen preferentially selects *creA* loss-of-function mutants resulting in sufficient expression of the *amdS* gene from the *faeB* promoter to allow growth on acetamide plates. To verify whether mutations in *creA* were responsible for the constitutive activation of the *faeB* promoter, the *creA* gene was sequenced in the 12 additional mutants in the JR10.2 background, and in 20 randomly picked mutants in the JR11.1 background. Among the 12 JR10.2-derived mutants, 10 mutants contain a mutation in the *creA* gene. Two mutants (JR10.2 U#92 and JR10.2 U#98) did not contain a mutation in the *creA* gene. ln the JR11.1 background, three of the 20 mutants (JR11.1 U#3, JR11.1 U#19 and JR11.1 U#79) do not contain a mutation in the *creA* coding region, whereas the remaining 17 mutants have a mutation in the *creA* gene. ln Fig. 5a and b, and Suppl. Table 2, the location of the mutations and the frequency of finding a certain *creA* allele in the mutants is shown. From the 27 mutants with mutations in *creA*, 22 mutants were found to have a mutation in either the first C2H2 motif (7 mutants) or the second C2H2 motif (15 mutants). Moreover, several alleles were found multiple times; e.g. the L113STOP or R117C mutations were found six and four times, respectively, in independently isolated mutants. The C78S mutation was found twice and three different amino acid changes were found at the C106 position (C106S, C106Y, and C106P) suggesting that there is a preference for certain mutations at certain positions in the *creA* gene. Five mutants contain small deletions or insertions in the *creA* gene leading to frame shifts (Fig. 5a). From the analysis, it became clear that our screen strongly selects for mutants with mutations in the DNA binding domain of CreA (25 out of 27 mutants). It is also clear from the analysis of the mutants that the two different genetic backgrounds used (JR10.2 and JR11.1) did not affect the outcome of the genetic screen. ln only two mutants (JR11.1 U#6 and JR10.2 U#80) the DNA binding motif is not mutated, but frame shift mutations occur after the DNA binding domains resulting in a non-functional protein. The growth phenotype on acetamide and allyl alcohol and the expression of luciferase under non-inducing and inducing conditions of these mutants was not different from the other mutants. The strong bias to isolate mutant in the DNA-binding motif strongly suggests that lack of binding of *creA* to the promoter is important to reach the state of inducer-independent expression from the *faeB* promoter. The 1 kb promoter of *faeB* contains 14 putative CreA binding sites (5’-SYGGRG-3’) and the lack of CreA binding to the promoter may have an important influence on the accessibility and binding of the transcription activation complex to the promoter.

### Targeted deletion of *creA* in the reporter strain results in ferulic acid-independent activation of the *faeB* promoter

**Fig. 6:**
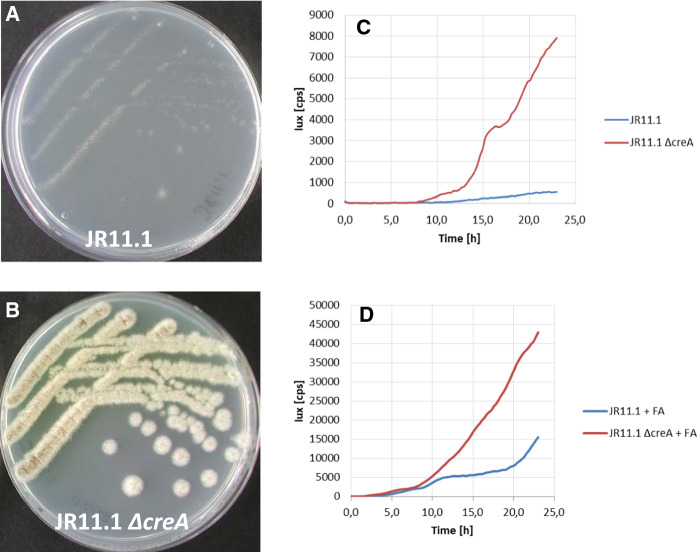
Deletion of *creA* in JR11.1 results in ferulic acid independent expression from the *faeB* promoter. **a** and **b** Strains JR11.1 and JR11.1 *ΔcreA* were grown on MM containing 1 % D-fructose and 10 mM acetamide at 30 °C for five days. **c** and **d** Lux assay was performed on strains JR11.1 and JR11.1 *ΔcreA*, using D-fructose as carbon source without (**c**) or with the addition of ferulic acid (FA) (**d**). Growth and luciferase activity were measured every 15 min for 24 h at 30 °C

To confirm that loss of function of *creA* is responsible for the constitutive activation of the *faeB* promoter, the *creA* gene was deleted in the JR11.1 reporter strain using split marker fragments (Arentshorst et al. 2015). Proper replacement of the *creA* gene by the phleomycin resistance marker was verified via diagnostic PCR (Supplementary Fig. 3). Subsequent growth analysis showed that deletion of *creA* in the JR11.1 reporter strain allowed the strain to grow on acetamide plates containing D-fructose without inducer (Fig. 6a and b). Deletion of *creA* in the dual reporter strain resulted in a 8-fold induction of luminescence under non-inducing conditions (Fig. 6c), confirming that inactivation of CreA leads to inducer-independent expression from the *faeB* promoter. We further addressed the question whether the expression from the *faeB* promoter was also affected by the absence of CreA when the inducer is present. As shown in Fig. 6d, luminescence was about four-times higher in the *ΔcreA* reporter strain compared to the reporter strain with intact *creA*, similar as observed for the mutants selected in our screen. This confirms that CreA represses the expression from the *faeB* promoter in the presence of both D-fructose and ferulic acid.

## Conclusions

Lignocellulosic biomass is considered to be the most favorable source for the production of second generation biofuels and other bio-based building blocks (Fatma et al. 2018; Poovaiah et al. 2014). Optimal enzyme cocktails are necessary for the efficient degradation of the lignocellulose into monosaccharides and aromatics. Ferulic acid is an important cross-linker in plant cell walls causing inefficient hydrolysis. To discover ways to enrich enzyme cocktails for enhanced presence of feruloyl esterases, this study was conducted to understand transcriptional regulation of feruloyl esterase B in *A. niger*. By identifying (transcription) factor(s) that control expression of *faeB*, approaches to modulate these transcription factors could lead to higher production of feruloyl esterases in cocktails (Alazi and Ram 2018). To search for transcription factors involved in *faeB* regulation a forward genetic screen was performed to obtain mutants with a constitutive expression from the *faeB* promoter. Many of the mutants contained a loss of function mutation in CreA, indicating that CreA plays an essential role in the repression of *faeB* on D-fructose. The detailed analysis of the mutants showed that most of the *creA* mutants carried mutations in the DNA binding motif, making them loss of function mutations. Although it has been reported that *faeB* expression is higher in the *creA* mutant (de Vries et al. 2002), this is the first study that shows that the deletion of *creA* leads to low but constitutive expression from the *faeB* promoter on D-fructose without an aromatic inducer. While a CreA loss-of-function leads to a low expression from the *faeB* promoter on D-fructose, addition of a strong inducer, such as ferulic acid, results in a much higher expression as has also been observed by de Vries et al. 2002. To find additional factors involved in the carbon catabolite related transcriptional control of *faeB*, the mutants that do show constitutive expression from the *faeB* promoter but have no mutation in *creA*, are currently being investigated further.

## Supplementary Information

Below is the link to the electronic supplementary material.

Supplementary material 1 (PPTX 97.6 kb)

Supplementary material 2 (PPTX 72.9 kb)

Supplementary material 3 (DOCX 278.9 kb)

Supplementary material 4 (DOCX 18.0 kb)

Supplementary material 5 (XLSX 17.8 kb)

Supplementary material 6 (XLSX 31.2 kb)

## Data Availability

The DNA reads described in this study are deposited in the Sequence Read Archive under accession number PRJNA623364. Other materials are available under request.
