# Supplementary material for: Loss of function of the carbon catabolite repressor CreA leads to low but inducer‐independent expression from the feruloyl esterase B promoter in Aspergillus niger
Source: Biotechnol Lett. 2021 Mar 18;43(7):1323–36. doi: 10.1007/s10529-021-03104-2 (PMC8197723; doi:10.1007/s10529-021-03104-2)
Supplement: Supplementary file 1 — Supplementary material 1 (PPTX 97.6 kb) [file 10529_2021_3104_MOESM1_ESM.pptx]

## Slide 1
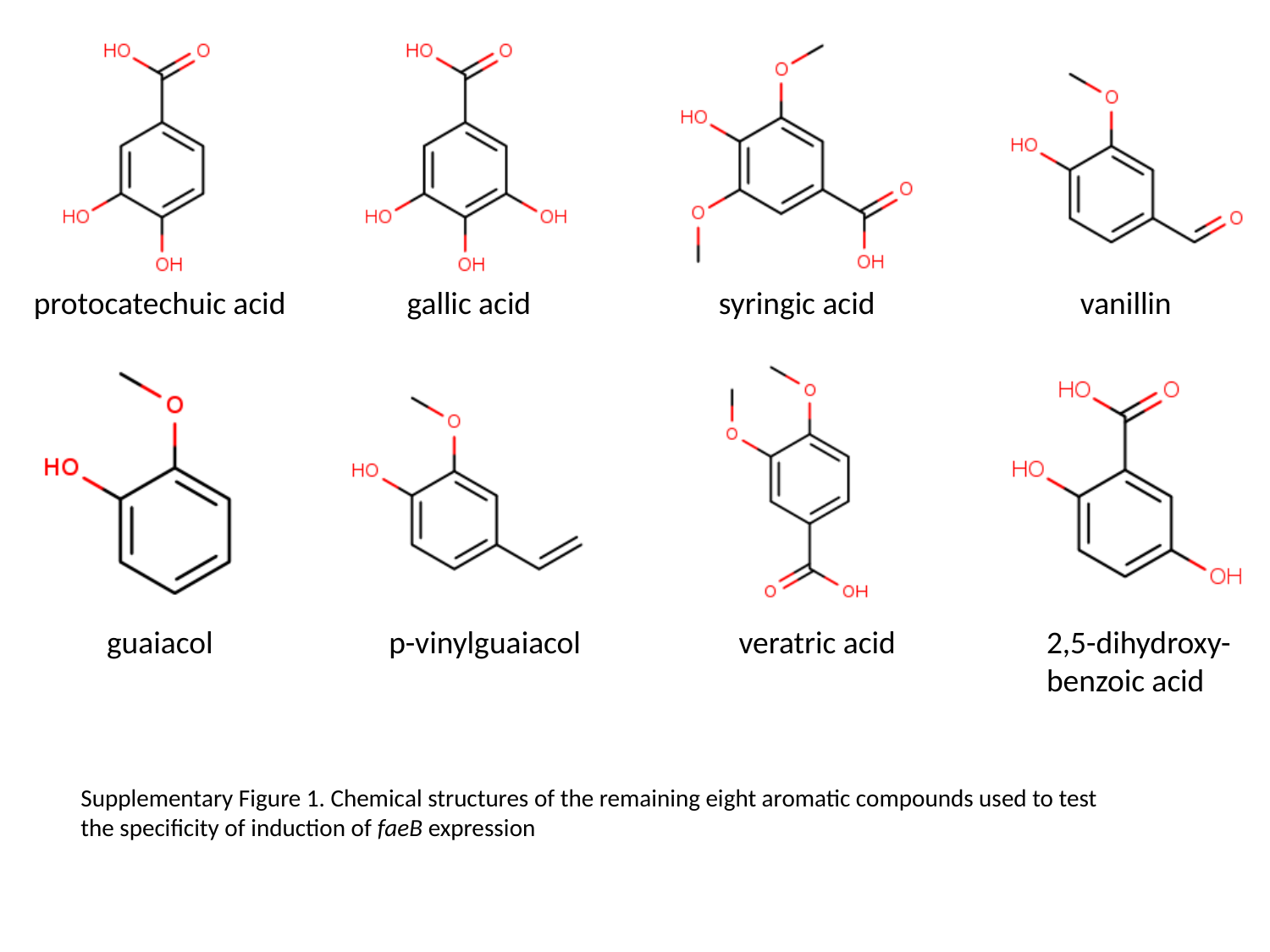

protocatechuic acid
gallic acid
syringic acid
vanillin
guaiacol
p-vinylguaiacol
veratric acid
2,5-dihydroxy-
benzoic acid
Supplementary Figure 1. Chemical structures of the remaining eight aromatic compounds used to test the specificity of induction of faeB expression
