# Supplementary material for: Loss of function of the carbon catabolite repressor CreA leads to low but inducer‐independent expression from the feruloyl esterase B promoter in Aspergillus niger
Source: Biotechnol Lett. 2021 Mar 18;43(7):1323–36. doi: 10.1007/s10529-021-03104-2 (PMC8197723; doi:10.1007/s10529-021-03104-2)
Supplement: Supplementary file 2 — Supplementary material 2 (PPTX 72.9 kb) [file 10529_2021_3104_MOESM2_ESM.pptx]

## Slide 1
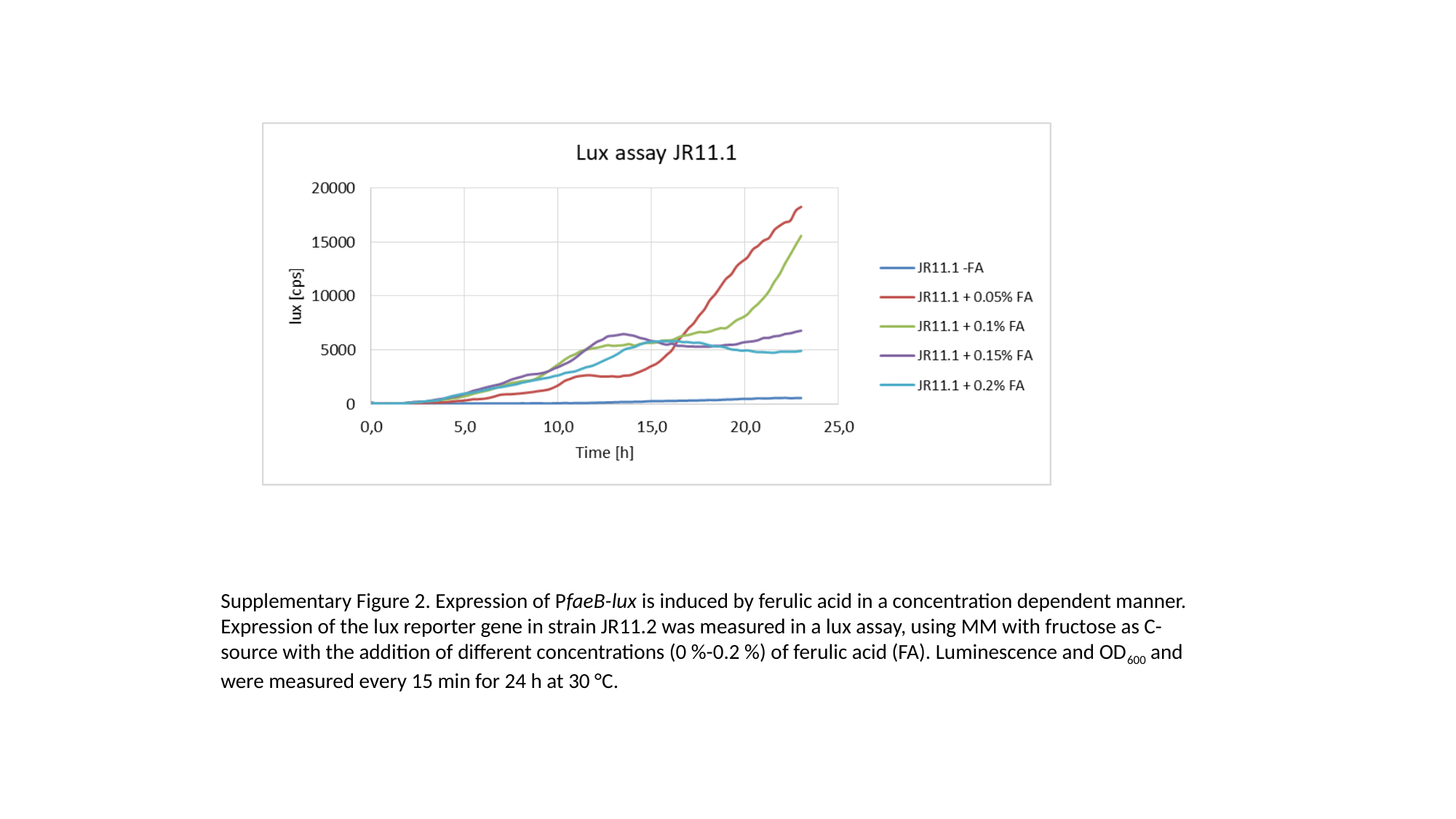

Supplementary Figure 2. Expression of PfaeB-lux is induced by ferulic acid in a concentration dependent manner. Expression of the lux reporter gene in strain JR11.2 was measured in a lux assay, using MM with fructose as C-source with the addition of different concentrations (0 %-0.2 %) of ferulic acid (FA). Luminescence and OD600 and were measured every 15 min for 24 h at 30 °C.
