# Supplementary material for: Loss of function of the carbon catabolite repressor CreA leads to low but inducer‐independent expression from the feruloyl esterase B promoter in Aspergillus niger
Source: Biotechnol Lett. 2021 Mar 18;43(7):1323–36. doi: 10.1007/s10529-021-03104-2 (PMC8197723; doi:10.1007/s10529-021-03104-2)
Supplement: Supplementary file 3 — Supplementary material 3 (DOCX 278.9 kb) [file 10529_2021_3104_MOESM3_ESM.docx]

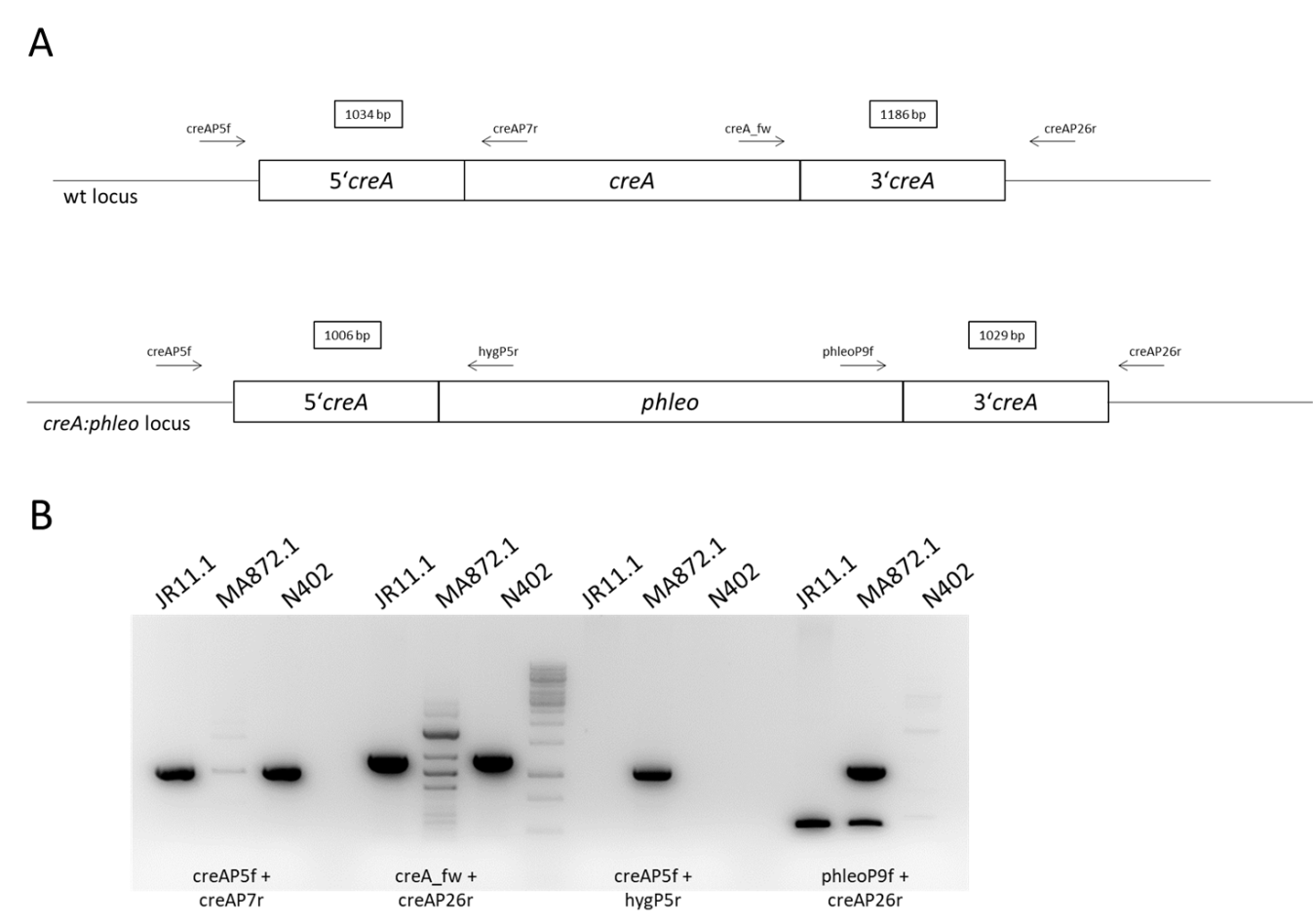


Supplementary Figure 4. Diagnostic PCR to verify *creA::phleo* deletion in JR11.1. A) Schematic representation of the wild type *creA* locus and the *creA::phleo* locus in the deletion strain. Annealing sites of the primers used are indicated together with the expected size of the resulting PCR products. B) Genomic DNA of a putative *creA::phleo* transformant (MA872.1), the recipient strain JR11.1 and N402 were used as template in the diagnostic PCR. Four different primer combinations were used. Strain MA872.1 was used for further analysis.
