# Supplementary material for: Loss of function of the carbon catabolite repressor CreA leads to low but inducer‐independent expression from the feruloyl esterase B promoter in Aspergillus niger
Source: Biotechnol Lett. 2021 Mar 18;43(7):1323–36. doi: 10.1007/s10529-021-03104-2 (PMC8197723; doi:10.1007/s10529-021-03104-2)
Supplement: Supplementary file 4 — Supplementary material 4 (DOCX 18.0 kb) [file 10529_2021_3104_MOESM4_ESM.docx]

Supplementary Table 1. Overview of the primers used in this study

| Primer name | Sequence (5’-3’) | Remark | Used for |
| --- | --- | --- | --- |
| faeBP1f_NotI | AAGGAAAAAAGCGGCCGCATGCCGATCGGTGATGACC | *Not*I site underlined | Amplification 997 bp *faeB* promotor |
| faeABP2r | GATGGCAAGCGGCGTGTGGTACCAGGAATTCCTTTGCAAGCTCAGAGTGGGAAC | Overlap with amdSP3f underlined | Amplification 997 bp *faeB* promotor |
| amdSP3f | GAATTCCTGGTACCACACGCC |  | Amplification 2.1 kb *amdS-TamdS* region |
| amdSP4r_NotI | GCGGCCGCTCTAGACTGGAAACGC | *Not*I site underlined | Amplification 2.1 kb *amdS-TamdS* region |
| PfaeB_PmeI_P5f | GTTTAAACATGCCGATCGGTGATGACC | *Pme*I site underlined | Amplification 997 bp *faeB* promotor |
| PfaeB_PmeI_P6r | GTTTAAACTTTGCAAGCTCAGAGTGGGAAC | *Pme*I site underlined | Amplification 997 bp *faeB* promotor |
| creA_sm_p1f | AAGCAGCCGATCTGGTTCAA |  | Amplification 5’ flank *creA* gene |
| creA_sm_p2r | CAATTCCAGCAGCGGCTTGTGAAGCTTGTCCCAAGAC | Overlap with phleoP4f underlined | Amplification 5’ flank *creA* gene |
| creA_sm_p3f | ACACGGCACAATTATCCATCGTTCGAACATTCTTCAGCCACAC | Overlap with phleoP5r underlined | Amplification 3’ flank *creA* gene |
| creA_sm_p4r | GGGAATGGTCTGGTCTCCGT |  | Amplification 3’ flank *creA* gene |
| phleoP4f | AAGCCGCTGCTGGAATTGCTCTTTCTGGCATGCGGAG | Overlap with creA sm p2r underlined | Amplification phleomycin split marker 5’ fragment |
| phleoP8r | GGAAGTTCGTGGACACGACC |  | Amplification phleomycin split marker 5’ fragment |
| phleoP5r | CGATGGATAATTGTGCCGTGTGGAGCATTCACTAGGCAACCA | Overlap with creA sm p3f underlined | Amplification phleomycin split marker 3’ fragment |
| phleoP6f | AAGTTGACCAGTGCCGTTCC |  | Amplification phleomycin split marker 3’ fragment |
| creAP5f | TGGGAAAGCTGACTGACCG |  | Diagnostic PCR to confirm *creA* deletion |
| creAP7r | CGACGCCATGTTGGAGTTC |  | Diagnostic PCR to confirm *creA* deletion |
| creA_fw | GTCATTTGTCGCTCCACCAC |  | Diagnostic PCR to confirm *creA* deletion |
| creAP26r | AGTACTGACTGACGATCGGCG |  | Diagnostic PCR to confirm *creA* deletion |
| hygP5r | ATCCACTGCACCTCAGAGCC |  | Diagnostic PCR to confirm *creA* deletion |
| phleoP9f | AGGACACACATTCATCGTAGGT |  | Diagnostic PCR to confirm *creA* deletion |
| p2f_creA_dPCR | TCGCGACGTG CATCACTATC |  | Amplification of creA ORF for sequencing |
| p3r_creA_dPCR | GACACCAATG ATGCCCATGA |  | Amplification of creA ORF for sequencing |
| creA_rev | TGTTCCGTCCGGTTTAGACA |  | Sequencing of creA ORF |
